# Effects of Selenium on Chronic Kidney Disease: A Mendelian Randomization Study

**DOI:** 10.3390/nu14214458

**Published:** 2022-10-23

**Authors:** Shaojie Fu, Li Zhang, Fuzhe Ma, Shuai Xue, Tao Sun, Zhonggao Xu

**Affiliations:** 1 Department of Nephrology, The First Hospital of Jilin University, Changchun 130021, China; 2 Department of Thyroid Surgery, General Surgery Center, The First Hospital of Jilin University, Changchun 130021, China

**Keywords:** chronic kidney disease, Mendelian randomization analysis, selenium, environmental factor, epidemiology

## Abstract

Background: Previous observational studies have shown that there is a controversial association between selenium levels and chronic kidney disease (CKD). Our aim was to assess the causal relationship between selenium levels and CKD using Mendelian randomization (MR) analysis. Methods: We used the two-sample Mendelian randomization (MR) method to analyze the causal role of selenium levels on CKD risk. The variants associated with selenium levels were extracted from a large genome-wide association study (GWAS) meta-analysis of circulating selenium levels (*n* = 5477) and toenail selenium levels (*n* = 4162) in the European population. Outcome data were from the largest GWAS meta-analysis of European-ancestry participants for kidney function to date. Inverse variance weighted (IVW) method was used as the main analysis and a series of sensitivity analyses were carried out to detect potential violations of MR assumptions. Results: The MR analysis results indicate that the genetically predicted selenium levels were associated with decreased estimated glomerular filtration (eGFR) (effect = −0.0042, 95% confidence interval [CI]: −0.0053–0.0031, *p* = 2.186 × 10^−13^) and increased blood urea nitrogen (BUN) (effect = 0.0029, 95% confidence interval [CI]: 0.0006–0.0052, *p* = 0.0136) with no pleiotropy detected. Conclusions: The MR study indicated that an increased level of selenium is a causative factor for kidney function impairment.

## 1. Introduction

Chronic kidney disease (CKD) can be identified by a decrease in estimated glomerular filtration (eGFR) rate (<60 mL/min/1.73 m^2^) persisting for at least three months [1]. CKD is a major global burden of disease and is recognized as an important risk factor for cancers and cardiovascular disease [2,3]. Globally, 697.5 million cases of CKD were reported in 2017 [3]. CKD and its effect on cardiovascular disease are responsible for 35.8 million disability-adjusted life years and 2.6 million deaths [3]. CKD has now become a global public health problem of great burden [4]. Selenium is an essential trace element for humans [5]. Recently, selenium has been reported to participate in the progression of CKD [6,7]. Due to its importance, selenium as a supplement has received increasing attention nowadays. Therefore, clarifying the relationship between selenium and CKD is very important.

Selenium is obtained through the diet. The form of selenium supplementation as well as the dose of selenium supplementation are both important for the health of organisms [8,9,10]. Cells need selenium for many processes, such as the antioxidant process, defense against infection and energy transfer [11,12]. Selenium exerts its effect largely through its presence in the active site of several biologically active selenoproteins [13,14,15]. Among them, selenium is best known in the form of glutathione peroxidase-3 (GPX-3), the strongest antioxidant enzyme in the body [14]. In addition, iodothyronine deiodinases and selenoprotein P have also been studied intensively [16,17,18]. The visceral organ with the highest distribution of selenium in the body is the kidney [19]. Most studies on the relationship between selenium and CKD are controversial [6,7,20,21,22,23,24,25,26,27,28,29,30,31]. Selenium levels are usually lower in CKD patients than normal people [25,26,27,28]. Several studies have found that adequate intake of selenium may exert positive effects on CKD [7,20]. However, some studies have also come to the opposite conclusion, indicating that selenium supplementation does not benefit patients with CKD and may even cause damage [21,23,24,30,31]. In fact, the sample size of these studies was small, and reverse causal inference cannot be avoided. Moreover, prospective cohort studies on selenium and CKD are scarce, so the causal relationship between selenium and CKD remains unclear.

Mendelian randomization (MR) analysis is a new and powerful epidemiological method to reinforce the random inference of causal effect on modifiable exposures (risk factors) by using genetic variation as an instrumental variable [32]. MR may be more plausible because the genetic variants are randomly assorted at conception and therefore can minimize residual confounding factors more than conventional methods [32,33]. Here, we used the two-sample Mendelian randomization method to explore the relationship between selenium and CKD, so as to provide a more comprehensive understanding of selenium’s function and guide the intake of selenium in patients with CKD.

## 2. Materials and Methods

### 2.1. Study Design

**Figure 1:**
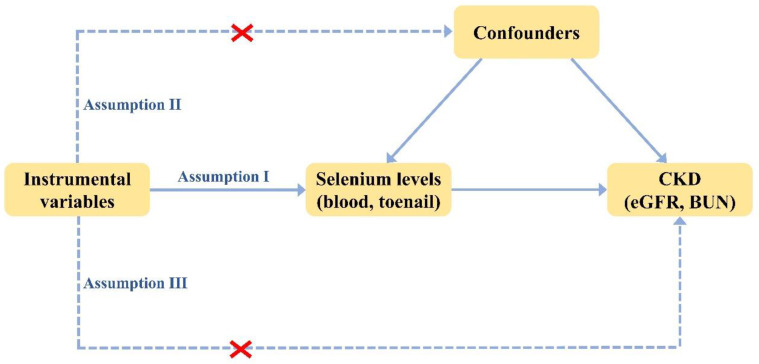
Three basic assumptions in MR analysis.

To investigate the causal relationship between the level of selenium and CKD, we performed MR analyses of selenium with eGFR (primary characteristics in assessing kidney function) and BUN (secondary characteristics in assessing kidney function), respectively. To ensure the reliability of the results, three basic assumptions must be met in every MR analysis [34]: (1) instrumental variables are solidly correlated with exposure, (2) instrumental variables are not related to any confounders influencing both exposure and outcome, (3) the influence of instrumental variables on outcomes is only via their effect on exposure rather than any other causal pathways (Figure 1).

### 2.2. Selenium Levels Exposure

The single nucleotide polymorphisms (SNPs) strongly correlated with selenium levels (*p* < 5 × 10^−8^) were obtained from a large genome-wide association study (GWAS) meta-analysis of blood and toenail selenium [35]. The concentrations of toenail selenium were gathered from 4162 European descendants in four U.S. cohorts (with genetic associations adjusted for age, sex, smoking status and study-specific covariates) [36,37,38,39]. The concentrations of blood selenium were gathered from 2874 pregnant women from the UK and 2603 Australian twins and their families (with genetic associations adjusted for age, gender and within-family relatedness) [40]. The 1000 Genomes Project linkage disequilibrium structure (r^2^ < 0.3 with any other associated SNP within 10 Mb) was tested among the initially selected SNPs to make sure that the selected instrumental variables were able to predict exposure independently. In addition, the proportion of variance (R2) and the F-statistics of the instrumental variables were also calculated. The SNPs with an F-statistic < 10 were identified as weak instruments and were excluded from instrumental variables [41]. All instrumental variables were searched in the database PhenoScanner V2 to evaluate whether they were significantly correlated with the risk factors for CKD and its subtypes [42].

### 2.3. Kidney Function Outcome

The summary-level data related to CKD involving measures of kidney function (i.e., eGFR and BUN) were obtained from the largest GWAS meta-analysis of European-ancestry participants for kidney function to date [43]. Reduced glomerular filtration rate is a defining parameter of CKD and could be estimated from serum creatinine levels [44]. Notably, serum creatinine may reflect more than just kidney function as a metabolite from muscle metabolism [45]. Therefore, more alternative kidney function biomarkers need to be considered, of which BUN is the most commonly used in clinical studies [46]. The eGFR GWAS meta-analysis includes the data from both the Chronic Kidney Disease Genetics (CKDGen) Consortium (*n* = 765,348) and UK Biobank (*n* = 436,561), and the GWAS meta-analysis for BUN was conducted in the CKDGen Consortium (*n* = 852,678). In CKDGen, GFR was estimated based on the CKD-EPI (for individuals > 18 years) and the Schwartz formula (for individuals ≤ 18 years) [47,48]. While, for all studies involved in UKB analysis, GFR was estimated using CKD-EPI [47].

The participants involved in these GWAS studies were all of European ancestry. For all studies, study participants gave informed consent and local ethics committees approved the study protocols.

### 2.4. Statistical Analysis

R software (version 4.1.1) with packages TwoSampleMR was used for all statistical analyses [49]. Since the estimated effects for blood and toenail selenium levels were represented in terms of Z-score units for each effect allele, the β and standard error values were converted from Z-score by the formulas reported by Taylor et al. [50] In order to conduct a comprehensive and precise investigation of causal effects, multiple complementary MR methods were employed, including the inverse variance weighted (IVW), the Mendelian randomization–Egger (MR-Egger) and the weighted median (WM). The IVW method combines exposure and outcome using correlations (β and standard error values) to regress each genetic variant in turn [51]. Following the convention in two-sample MR studies, it is used as the fundamental estimates of the causal effects of exposures on outcomes [52]. Heterogeneity was illustrated using a random effect model. As the IVW method uses summarized data from all the genetics variants, whereas the WM method only requires most variants to be valid instruments, the WM method was included as a complementary test [53]. MR-Egger provides an assessment of the underlying asymmetry for the pleiotropic effects of multiple genetic variants [54]. Therefore, MR-Egger was conducted to consider the bias resulting from the directional horizontal pleiotropy. Leave-one-out (LOO) analysis and Mendelian randomization pleiotropy residual sum and outlier (MR-PRESSO) were performed to identify the outliers, with a significant influence on causal effect [55].

### 2.5. Power Analysis

To assess the minimum detectable magnitude of outcomes in the causal relationship of selenium levels and CKD, the statistical power of this bidirectional MR was conducted using a web application named mRnd [56].

## 3. Results

### 3.1. Development of the Selenium Levels Genetic Instruments

A total of 11 independent, non-palindromic and significant SNPs (*p* < 5 × 10^−8^) were selected as instrumental variables to genetically predict the selenium levels. The 11 SNPs explained 0.32–1.76% of the variance in selenium levels and the F statistics for these genetic instruments were all larger than 10, indicating the instruments were strong. In addition, all of these instrumental variables are located on chromosomes 5 and 21, and multiple SNPs are located near the DMGDH, CBS and betaine-homocysteine S-methyltransferase (BHMT) genes (Table 1). In the instrumental variables we identified, the SNP rs921943 is strongly correlated with the outcome eGFR, which violates the basic assumption III in the MR analysis, so the SNP rs921943 is excluded from the instrumental variables in the MR analyses of selenium with eGFR.

### 3.2. Association of Selenium with eGFR

**Figure 2:**
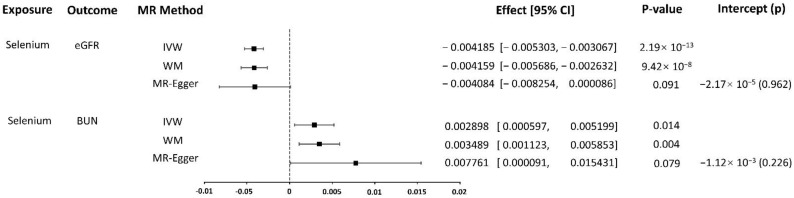
MR results.

We used the IVW method to evaluate the association of selenium levels-associated SNPs and eGFR risk. No heterogeneity of effects was detected through Cochran’s Q test (*p* = 0.429). This conventional MR analysis showed an association between selenium levels and eGFR risk (effect = −0.0042, 95% confidence interval [CI]: −0.0053–−0.0031, *p* = 2.186 × 10^−13^). WM method as a complementary test also obtained the consistent conclusion that selenium levels were strongly associated with lower eGFR (effect = −0.0042, 95% CI: −0.0057–−0.0026, *p* = 9.416 × 10^−8^). Although the association estimates from sensitivity analysis using MR-Egger were not statistically significant (*p* = 0.0911), their direction was negative, consistent with the other analyses (Figure 2). In the causality investigated, MR-Egger did not detect evidence of pleiotropy, since the P value for its intercept was >0.05 (*p* = 0.9617). No outliers were identified with MR-PRESSSO and the leave-one-out plot (Figure S1).

### 3.3. Association of Selenium with BUN

We used the IVW method to evaluate the association of selenium levels-associated SNPs and BUN risk. There was a heterogeneity of effects detected through Cochran’s Q test (*p* = 0.043). As we used random-effects IVW as the main result, the heterogeneity is acceptable [57]. The random-effects IVW analysis showed an association of selenium levels with BUN risk (effect = 0.0029, 95% CI: 0.0006–0.0052, *p* = 0.0136). WM method as a complementary test also obtained the consistent conclusion that selenium levels were strongly associated with higher BUN (effect = 0.0035, 95% CI: 0.0011–0.0059, *p* = 0.0038). Although the association estimates from sensitivity analysis using MR-Egger were not statistically significant (*p* = 0.0787), its direction was positive, consistent with the other analyses (Figure 2). In the causality investigated, MR-Egger did not detect evidence of pleiotropy, since the P value for its intercept was >0.05 (*p* = 0.9617). No outliers were identified with MR-PRESSSO, but in the leave-one-out plot, we could find that the overall IVW estimate of all SNPs associated with selenium level on the risk of BUN was significantly affected by two SNPs rs921943 and rs10944 (Figure S2).

### 3.4. Power

Considering the sample size and the variance in selenium levels explained by the instrumental variables we identified, we used mRnd to evaluate the statistical power of this MR study. Power calculations showed that our study had at least 99% power to detect the beta value of −0.0042 for eGFR and 0.0029 for BUN per standard deviation of selenium levels.

## 4. Discussion

The relationship between selenium levels and kidney function has been unsettled for years. Conventional observational studies have found that patients with CKD usually have lower selenium levels in tissue (whole blood and hair), plasma and serum than in the healthy population [25,26,27,28]. Furthermore, selenium levels have been found to correlate with the severity of CKD; as CKD progresses and eGFR decreases, plasma levels of selenium in patients also decrease [6,29]. A recent cross-sectional study based on the data from the China Health and Nutrition Survey (CHNS) found that an adequate selenium intake may have a positive effect on CKD [20]. Controversially, another cross-sectional study in elderly people aged ≥ 90 years did not find a correlation between selenium and CKD [21]. In addition, some studies have found no beneficial effects of selenium supplementation on lipid profiles, thyroid function tests or acute phase reactants in CKD patients on hemodialysis [23,24]. Furthermore, a large randomized, double-blind controlled clinical trial in Denmark showed long-time high doses of selenium intake would increase the mortality rate accordingly [30]. Therefore, low selenium levels and CKD appear to be causally related to each other, but there is no clear clinical evidence that selenium deficiency causes CKD [31]. Since traditional observational studies are susceptible to reverse causation and confounding, it remains to be clarified whether selenium levels play a delaying or promoting role in the development of CKD.

To our knowledge, this is the first study to explore the causal relationship between selenium levels and risk of CKD, using MR methods. The greatest advantage of MR methods is the use of genetic variation as proxies for each trait. Since people with selenium-raising and selenium-lowering genetic variants have been exposed to these variants since conception, reverse causation can be avoided [58]. Confounding can be mitigated by eliminating the genetic variants associated with confounders and do not exhibit pleiotropic effects [59]. The reliability of MR results depends on three basic assumptions, but they could be breached by population stratification, canalization and pleiotropy [60]. We limited the study group to European ancestry individuals to minimize the population stratification. Canalization is the compensatory processes that alleviates genetic effects during development. Although the influence of canalization on results cannot be directly tested, it would tend to make the results biased toward null and thus fail to explain the observed association of selenium levels with eGFR and BUN. As for pleiotropy, we performed some sensitivity analyses to evaluate and adjust it. In the sensitivity analyses, the association of genetically predicted selenium levels concentrations with eGFR and BUN was robust, and no directional pleiotropy was found in the MR-Egger analysis. Notably, blood selenium levels are sensitive to recent selenium exposure, reflecting approximately 17 weeks of exposure, while selenium levels in toenails reflect longer exposure, approximately 26–52 weeks [35]. Therefore, to better reflect the selenium exposure, we selected the GWAS study that included both blood and toenail selenium levels. Additionally, our study incorporated the summary statistics of selenium levels and kidney function measurements from large-scale cohorts of European ancestry, with great statistical power, so the inference was credible.

Interestingly, all of these instrumental variables solidly correlated with selenium levels are located on chromosomes 5 and 21, and multiple SNPs are located near the DMGDH, CBS and BHMT genes. A recent GWAS study in European populations found that alleles of 2 SNPs in the DMGDH region and 2 SNPs in the BHMT region were associated with increased selenium concentrations following selenium supplementation [61], which further supports the importance of the variants in the DMGDH and BHMT regions of chromosome 5 on selenium metabolism. Both the dimethylglycine dehydrogenase encoded by DMGDH and the betaine-homocysteine S-methyltransferase encoded by BHMT have been demonstrated to be implicated in homocysteine metabolism [62,63]. It has been also reported that selenium concentration is negatively correlated with homocysteine concentration in the blood [64]. These suggest a possible link between selenium exposure and the homocysteine metabolic pathway. Cystathionine β-synthase encoded by CBS is an important enzyme that catalyzes the generation of hydrogen sulfide [65]. Metabolism of selenium in vivo could also be coupled with the trans-sulfuration pathway. Seale et al. found that when selenoprotein P is disrupted, the available selenium in the liver increases, thereby activating the trans-sulfuration pathway to maintain selenium supply and facilitate selenoprotein production [66].

Our study observes strong evidence supporting the association of genetically predicted selenium levels with decreased eGFR and increased BUN, which gives a novel insight, that an increased level of selenium is a causative factor for kidney function impairment. In recent years, selenium supplementation has received increasing attention as a trace element with extremely important physiological functions in the antioxidant process, energy transfer and defense against infection [11,12]. Therefore, the results we observed are very interesting and important, suggesting that the supplementation of selenium should be considered more carefully, especially in people with renal insufficiency.

Selenium has no biological activity of its own, it exerts its effect largely through its presence in the active site of several biologically active selenoproteins [13,14,15]. Thus, selenoproteins, as selenium-dependent proteins, are carriers of the biological effects of selenium. Iodothyronine deiodinases, selenoprotein P and glutathione peroxidase are the most intensively studied selenoproteins. Iodothyronine deiodinases consists of three different selenium-dependent enzymes, which catalyzes the removal of an iodine residue from the thyroxine molecule, converting it into triiodothyronine or inactive metabolites [16]. A reduction in iodothyronine deiodinases leads to a reduction in peripheral triiodothyronine and a decrease in plasma triiodothyronine is strongly associated with inflammation in patients with CKD [17]. Selenoprotein P is an antioxidant protecting endothelial cells from damage induced by peroxynitrite [18]. Glutathione peroxidase is an antioxidant enzyme that protects vascular and immune cells from oxidative damage by reducing reactive oxygen species, such as superoxide anions and peroxides, to reduce oxidative stress, the best known of which is GPX3 [14,67]. Therefore, selenium seems to play a protective role against renal impairment.

However, there are at least 30 selenoproteins in humans and the biology for most of them, especially their effect on the kidneys is unclear [68]. There are some selenoproteins with potentially harmful effects, such as GPX1, which is associated with insulin resistance and type 2 diabetes [69,70]. Moreover, the beneficial and harmful effects of selenium depend on its dose and form [71]. As selenium intake increases and selenoprotein expression becomes saturated, the remaining selenium will be present primarily in nonspecific selenium-containing proteins [72]. Harmful effects occur when the non-specific form of selenium is incorporated nonspecifically into body proteins via selenomethionine (SeMet) in place of methionine [73]. SeMet can be metabolized to selenols/selenates via the methionine cycle and the trans-sulfuration pathway. Selenols/selenolates can undergo redox cycling, generating superoxide radicals and reacting with thiols/diselenides to produce selenyl sulphides/ disulphides, leading to protein aggregation, inactivation of transcription factors, disruption of redoxregulated cell signaling, and endoplasmic reticulum stress [30,74]. The supplemented form of selenium is extremely important for the health of organisms, and even more important than the dose of supplementation [8]. Selenium is generally bioavailable in both organic and inorganic forms. Inorganic compounds such as selenate and selenite account for a much smaller proportion of the dietary sources of selenium, while organic compounds such as L-selenocysteine and L-selenomethionine and their derivatives are the main sources of dietary selenium [9]. The organic form of selenium has a higher level of bioavailability compared to the inorganic form of selenium, which is 90–95% and 80–85%, respectively [10]. The inorganic form of selenium, namely selenate and selenite, could be efficiently reduced in living organisms and then used for the synthesis of selenoproteins. Notably, despite lower bioavailability, the inorganic selenium has a higher level of toxicity than the organic selenium [8]. The occurrence of selenosis is often related to excessive concentrations of selenium from exposure to the environment, or from the food consumed. Importantly, the measuring of selenium concentration includes not only functional selenium-containing proteins but also non-functional selenium compounds such as non-protein bound selenium and nonspecific selenium-containing proteins as SeMet [75]. Approximately 53% and 39% of the selenium in blood exists in selenoprotein P and GPX3, respectively, while the remainder is mainly present in non-functional selenium compounds [35]. Since selenoprotein P and GPX3 are fully expressed in selenium sufficient individuals, a change in selenium level is almost exclusively limited to change in non-functional selenium compounds; and the proportion of non-functional selenium compounds in the total content is also influenced by nutritional status [76]. Selenium deficiency, or excess, is therefore a relative concept. Patients with CKD are characterized by a reduced dietary intake, impaired intestinal absorption, increased urinary excretion of protein and decreased ability to synthesize selenoproteins. As a result, they are more prone to selenoprotein saturation when supplementing with selenium and therefore more susceptible to the toxic effects described above [26,77]. Furthermore, in patients with CKD, measured selenium levels are more difficult to reflect possible changes in selenium biochemistry and thus indicate changes in their health status. These are the possible mechanisms supporting our findings. Indeed, the pathogenesis of CKD is complex and further studies are still needed to elucidate the potential mechanisms linking selenium levels and CKD.

Given the potential toxicity of selenium in current study, we recommend that selenium supplementation should be carried out with great caution, especially in people with CKD. Waiting for the results of well-designed and adequately powered clinical trials to be reported is essential. It is possible that our study could be an inexpensive and time-efficient first step to predict the efficacy and possible adverse effects of a randomized controlled trial before it is designed.

There were several limitations in our study. First, the genetic analysis was based on individuals with European ancestry, so it is uncertain whether the results can be generalized to other ethnic populations, especially for selenium, an element whose levels in populations are strongly influenced by variations in geographical distribution. Second, our study only genetically predicted a causal effect between selenium levels and renal dysfunction and did not directly understand the underlying mechanisms of the adverse effects of increased selenium levels on renal function. Third, our MR relied on publicly available summary statistics, and we cannot obtain the original clinical result data of each individual, therefore, further analysis by population stratification was not possible. Fourth, as the GWAS study on selenium that we included measured overall levels of selenium and lacked stratified data on the levels of different forms of selenium, particularly the selenoproteins that exert biological activity, this may bias the results of the analysis, as these factors were significantly associated with clinical outcomes. Finally, as in all MR analyses, potential sample overlap may also have an impact on our results.

## 5. Conclusions

This MR study suggests the causal relationship between selenium levels and renal function. Elevated selenium levels may be a causal risk factor for decreased eGFR and increased BUN, which gives a novel insight, that an increased level of selenium is a causative factor for kidney function impairment. However, further studies and clinical trials are needed to investigate the potential underlying mechanisms of association and clinical relevance between selenium and renal function, and to further confirm these results by using genetic and environmental approaches.

## Figures and Tables

**Table 1:** The instrumental variables used to genetically predict the selenium levels.

SNP	Nearby Gene	Chr	E/O Allele	EAF	Association with the Exposure						Association with the Outcome: eGFR			Association with the Outcome: BUN		
					Beta	SE	*p* Value	Z-Score	K	R2	Beta	SE	*p* Value	Beta	SE	*p* Value
rs672413	ARSB	5	A/G	0.32	0.164418	0.021835	5.21 × 10^−14^	7.53	114.728	0.011765	−0.0012	3.00 × 10^−4^	6.91 × 10^−5^	6.00 × 10^−4^	5.00 × 10^−4^	0.2719
rs705415	DMGDH	5	T/C	0.14	−0.20006	0.032113	4.64 × 10^−10^	−6.23	78.36179	0.009638	8.00 × 10^−4^	4.00 × 10^−4^	0.0541	−4.00 × 10^−4^	8.00 × 10^−4^	0.6377
rs3797535	DMGDH	5	T/C	0.08	0.298102	0.037544	2.05 × 10^−15^	7.94	127.7319	0.013081	−8.00 × 10^−4^	5.00 × 10^−4^	0.1122	9.00 × 10^−4^	0.001	0.3606
rs11951068	DMGDH	5	A/G	0.07	0.268264	0.03992	1.86 × 10^−11^	6.72	91.15215	0.00937	−0.0014	5.00 × 10^−4^	0.008477	−2.00 × 10^−4^	8.00 × 10^−4^	0.7902
rs921943	DMGDH	5	T/C	0.29	0.294952	0.022447	1.90 × 10^−39^	13.14	358.0757	0.035825	−0.0021	3.00 × 10^−4^	2.62 × 10^−12^	0.001	5.00 × 10^−4^	0.05763
rs10944	BHMT2	5	T/G	0.49	0.257746	0.020375	1.13 × 10^−36^	12.65	330.9678	0.033203	−0.0012	3.00 × 10^−4^	6.42 × 10^−6^	0.0012	5.00 × 10^−4^	0.01758
rs567754	BHMT	5	T/C	0.34	−0.19588	0.021502	8.38 × 10^−20^	−9.11	168.8575	0.01722	8.00 × 10^−4^	3.00 × 10^−4^	0.005016	−0.0016	5.00 × 10^−4^	3.21 × 10^−3^
rs6859667	HOMER1	5	T/C	0.96	−0.35969	0.051978	4.40 × 10^−12^	−6.92	96.71387	0.009936	0.001	7.00 × 10^−4^	0.1318	−0.0017	0.0013	0.1955
rs6586282	CBS	21	T/C	0.17	−0.15971	0.027116	3.96 × 10^−9^	−5.89	69.87277	0.007198	0.0011	4.00 × 10^−4^	0.00323	4.00 × 10^−4^	7.00 × 10^−4^	0.5388
rs1789953	CBS	21	T/C	0.14	0.162035	0.029354	3.40 × 10^−8^	5.52	61.31581	0.006322	−2.00 × 10^−4^	4.00 × 10^−4^	0.6217	−0.0018	8.00 × 10^−4^	0.02094
rs234709	CBS	21	T/C	0.45	−0.11957	0.020474	5.23 × 10^−9^	−5.84	68.68309	0.007077	1.00 × 10^−4^	3.00 × 10^−4^	0.7589	4.00 × 10^−4^	5.00 × 10^−4^	0.4208

## Data Availability

Publicly available datasets were analyzed in this study. This data can be found here: https://pubmed.ncbi.nlm.nih.gov/25343990/ and https://pubmed.ncbi.nlm.nih.gov/34272381/.

## Supplementary Materials

The following supporting information can be downloaded at: https://www.mdpi.com/article/10.3390/nu14214458/s1, Figure S1: The leave-one-out plot of the overall IVW estimate of all selenium levels-related SNPs on the risk of eGFR, Figure S2: The leave-one-out plot of the overall IVW estimate of all selenium levels-related SNPs on the risk of BUN.
