# Supplementary material for: Effects of Selenium on Chronic Kidney Disease: A Mendelian Randomization Study
Source: Nutrients. 2022 Oct 23;14(21):4458. doi: 10.3390/nu14214458 (PMC9654848; doi:10.3390/nu14214458)
Supplement: Supplementary file 1 [file nutrients-14-04458-s001.zip › nutrients-1963959-supplementary.pdf]

Supplementary Material

Supplementary Figures

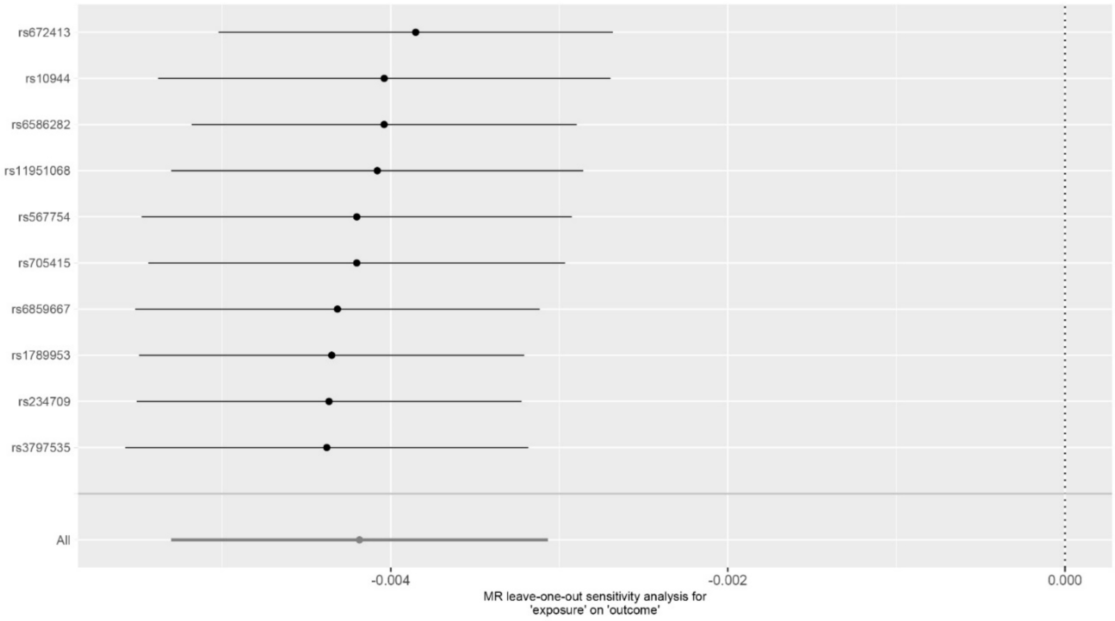

Supplementary Figure S1. The leave-one-out plot of the overall IVW estimate of all selenium levels-related SNPs on the risk of eGFR.

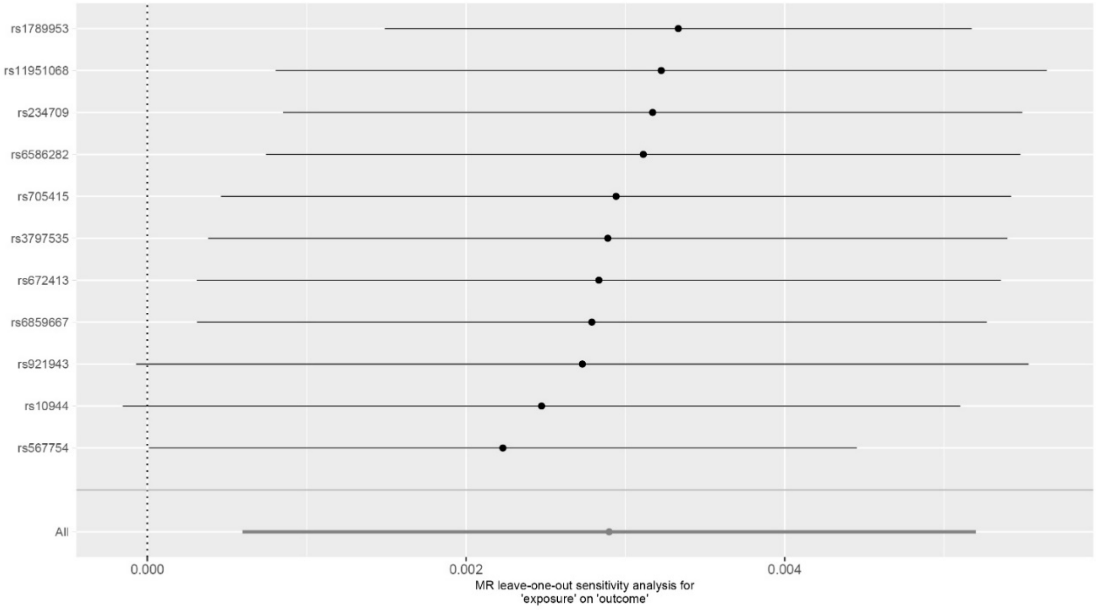

Supplementary Figure S2. The leave-one-out plot of the overall IVW estimate of all selenium levels-related SNPs on the risk of BUN.
